# The Protective Effects of α-Lipoic Acid on Kidneys in Type 2 Diabetic Goto-Kakisaki Rats via Reducing Oxidative Stress

**DOI:** 10.3390/ijms14046746

**Published:** 2013-03-26

**Authors:** Bo Feng, Xin-Feng Yan, Jun-Li Xue, Lei Xu, Hua Wang

**Affiliations:** Department of Endocrinology, Shanghai East Hospital, Tongji University School of Medicine, Shanghai 200120, China; E-Mails: xinfengyan@hotmail.com (X.-F.Y.); caorenyi_2000@hotmail.com (J.-L.X.); shirley_ok1@126.com (L.X.); tjwh02@163.com (H.W.)

**Keywords:** antioxidant, α-lipoic acid, diabetic nephropathy, oxidative stress

## Abstract

To evaluate the protective effects of α-lipoic acid on the kidneys of Goto-Kakisaki (GK) diabetic rats, ten GK diabetic rats were randomly divided into a diabetic control group and a lipoic acid-treated diabetic group with α-lipoic acid 35 mg·Kg^−1^ intraperitoneal injections. Four healthy Wistar rats served as normal controls. Malonaldehyde (MDA), ascorbic acid (vitamin C), vitamin E, glutathione (GSH) and superoxide dismutase (SOD) levels in renal homogenate, and urine protein excretion were measured. The expression of mRNA for NF-κB, NADPH oxidase subunits p22phox and p47phox in renal tissue was examined by realtime PCR. Pathological changes in renal tissue were evaluated by light and electron microscopy. There were significant increases in urine protein excretion, MDA levels and the expression of mRNA of NF-κB, p22phox and p47phox, and significant decreases in GSH, SOD, vitamin C and vitamin E levels in the diabetic control group compared with the normal control group. Pathological changes of renal tissue were more progressive in the diabetic control group than in the normal control group. All the parameters above were improved in the α-lipoic acid-treated diabetic group. Oxidative stress is increased in the kidney of type 2 diabetic GK rats. It is associated with the progression of diabetic nephropathy. α-lipoic acid can protect renal function in diabetic rats via its antioxidant activity.

## 1. Introduction

Diabetic nephropathy is a severe, chronic complication of type 2 diabetes mellitus, and it is the main cause of end-stage renal failure in diabetic patients [1]. Hyperglycemia results in an increased production of reactive oxygen species (ROS) which leads to renal dysfunction [2].

Oxidative stress is the “common soil” of the chronic complications associated with diabetes mellitus [3]. The increased oxidative stress leads to injuries of the glomeruli [4], tubular interstitial tissue [5] and vasculature [6]. It is implicated in the mesangial expansion of extra-cellular matrix, and results in increases in glomerular filtration rate, urine protein excretion, progression of glomerular sclerosis and tubular-interstial fibrosis [7–9]. Antioxidative therapy may be an effective way to treat diabetic nephropathy [10–12]. Recently, α-lipoic acid (ALA) was suggested as an effective antioxidant. ALA quenches singlet oxygen, hydroxyl radicals, hypochloric acid, superoxide anion radicals, peroxyl radicals and hydrogen peroxide. ALA has metal-chelating activity and it is able to regenerate other natural antioxidants, such as vitamin C or vitamin E from their radical or inactive forms [13,14]. In light of the antioxidant activity of ALA, the purpose of this study was to verify oxidative stress in the pathogenesis of diabetic nephropathy in type 2 diabetic rats and to evaluate the protective effect of ALA on diabetic nephropathy.

## 2. Results

### 2.1. Blood Glucose and Body/Kidney Weight Determination

**Figure 1:**
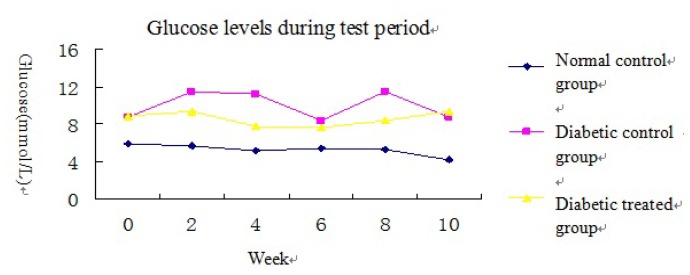
Glucose levels during test period.

Compared to the normal control group, blood glucose levels were significantly increased in the two diabetic groups however there was no significant difference in blood glucose levels between the diabetic control group and the ALA-treated diabetic group (Figure 1 and Table 1). Compared to the normal control group, body weight was decreased in the diabetic control group. Compared to the diabetic control group, body weight was greater in the ALA-treated diabetic group (Table 1). Kidney weight and kidney/body weight ratio increased significantly in the diabetic group compared to the normal control group. ALA treatment resulted in significant reduction in both kidney weight (*p* < 0.05) and kidney/body weight ratio (*p* < 0.05) compared to the diabetic control group (Table 1).

Figure 1 Blood glucose levels during test period in the three groups: the blood glucose levels of the two diabetic groups were significantly higher than that of the normal control group. The glucose level in the ALA treated diabetic group was a little lower than in the diabetic control group but did not reach significance.

### 2.2. Urine Protein Excretion (UPE)

UPE was increased significantly in the diabetic control group compared to the normal control group and was decreased in the ALA-treated group compared to the diabetic control group (Table 2).

### 2.3. Morphology and Realtime Reverse Transcriptase (RT)-PCR

The kidneys of rats in the normal control group showed normal cortical structure as determined by light microscopy. Compared to the normal control group, the index of glomerulosclerosis in the diabetic control group increased significantly. The index of glomerulosclerosis in the ALA-treated diabetic group was lower than in the diabetic control group (Table 2).

The area of the glomeruli (SG) increased significantly in the diabetic group compared to the normal control group. In contrast, the area of the glomeruli in the ALA-treated group was lower than in the diabetic control group. Similarly, the area of mesangial region (SM) in the diabetic control group increased significantly compared to the normal control group. The ALA-treated group decreased significantly compared to the diabetic control group (Table 2).

The thickness of the glomerular basement membrane (GBM) in the diabetic control group was higher than in the normal control group (*p* < 0.01), while the thickness of the GBM in ALA-treated group was lower than in the diabetic control group (*p* < 0.01) (Table 2).

**Figure 2:**
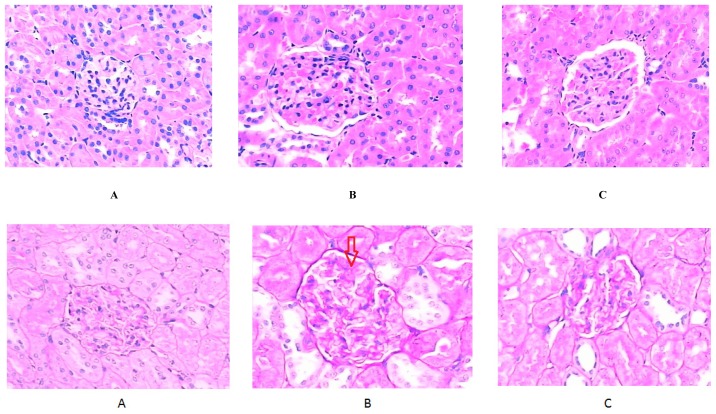

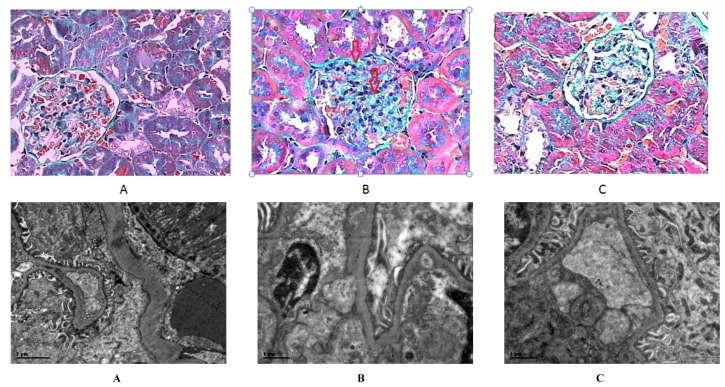
Histomorphologic changes verified by micro and ultraminiature observation of the kidneys: first line: hematoxylin and eosin (H&E) stain, ×800; second line: periodic acid-Schiff (PAS) stain, ×800; third line: Masson’s stain, ×800; Fourth line: electron microscopy; A. normal control group; B. diabetic control group; C. diabetic α-lipoic acid (ALA)-treated group. Glomerular hypertrophy, expansion of the mesangial region, mesangial cell proliferation, and some inflammatory cell infiltrate were also observed in H&E-stained sections under light microscopy. The expansion of the mesangial region, thickening and interruptions in the glomerular basement membrane (GBM) were all observed in PAS-stained sections. In Masson’s-stained sections of kidney tissue, the degree of collagen fiber stain was significantly increased in the diabetic control group, and decreased in the ALA-treated group.

As illustrated in Figure 2, the kidneys of the normal control group showed normal cortical morphological structure with consecutive and uniform glomerular basement membrane and a normal mesangial area when observed by light microscopy. Mild glomerulosclerosis, characterized by GBM thickening, mesangial expansion, intercapillary cell proliferation and increased extracellular matrix (ECM) was observed in the diabetic control group. Moreover, a diminished number of foot processes in some glomeruli was also observed. On electron microscopy GBM thickening and extension into capillary lumens was noted in the diabetic control group in addition to a disappearance of the windows between the capillary endothelium, widening of the foot process of the podocytes and also the ECM was significantly increased. Treatment with ALA alleviated glomerulosclerosis in the diabetic treated group with pathological changes mentioned above alleviated.

The mRNA expression of NF-κB, p22phox and p47phox increased significantly in the diabetic control group compared to the normal control group (*p* < 0.05), and decreased in the ALA-treated group compared to the diabetic control group (Table 3).

### 2.4. Biochemical Analysis

The levels of all antioxidants detected in this study (GSH, SOD, vitamin E, and vitamin C) were decreased significantly in the diabetic control group compared to the normal control group. In the ALA-treated group, the levels of these antioxidants increased significantly compared to the diabetic control group (Table 4).

The level of MDA, which reflects the level of lipid oxidation, was increased in the diabetic control group compared to the normal control group and treatment with ALA decreased the level of MDA.

### 2.5. Correlation Analyses

Correlation analyses showed that there were significant negative correlations between 24 h urine protein excretion rate and the levels of SOD (*r* = −0.537, *p* < 0.05), GSH (*r* = −0.823, *p* < 0.01), vitamin E (*r* = −0.645, *p* < 0.05). There was significant positive correlation between MDA levels and the mRNA expression of p22phox (*r* = 0.66, *p* < 0.01), p47phox (*r* = 0.665, *p* < 0.01), NF-κB (*r* = 0.641, *p* < 0.05) in renal tissue. mRNA expression of p22phox and p47phox was associated positively with NF-κB (*p* < 0.01). SG and SM were significantly negative associated with SOD level (*r* = −0.591 and −0.578, *p* < 0.05, respectively), and positively with the mRNA expression of p22phox (*r* = 0.587, *p* < 0.05 and 0.468, *p* = 0.091), NF-κB (*r* = 0.662, *p* < 0.01 and 0.538, *p* < 0.05).

## 3. Discussion

Oxidative stress exists in diabetes mellitus and is associated with dysfunction of the endothelium [15]. The diabetes animal model presented in this study confirmed that the level of oxygen free radicals was associated with both glomerulosclerosis and the expansion of the mesangial region. Supplementation with 35 mg/kg α-lipoic acid for 12 weeks prevented the increase of proteinuria in the diabetes group, and lessened renal pathological changes which are associated with diabetic nephropathy. In particular, ALA reduced the expression of NF-κB, NADPH oxidase subunits p22phox and p47phox, which play an important role in the generation of oxygen free radicals [16,17]. This study verified that one of the mechanisms by which ALA exerts this renal protective effect is via decreasing oxidative stress.

Previous studies in animal models have reported that the renal protective effects of ALA were predominantly achieved by reducing glycemia [7]. In the present study, supplementation with ALA for 12 weeks had some potential effect on ameliorating the blood glucose level but there was no significant decrease in blood glucose levels however the index of oxidative stress was greatly reduced. These results suggest that ALA’s renal protective effect is mainly through reducing the oxidative tissue damage rather than affecting the blood glucose level [17].

In diabetes, ROS is generated from an excess shunting of glucose going through the polyol and glucosamine pathways, which leads to the formation and activation of protein kinase C, formation of AGEs and glycation. This causes tissue damage by increased synthesis of ECM proteins and enhanced expression of inflammatory mediators, which contributes to glomerulosclerosis and tubulointerstitial fibrosis in kidney [18]. Thus, antioxidants therapy may play an important role to manage diabetic nephropathy.

GSH is one of the most abundant antioxidants in mammalian cells. As demonstrated in this study, ALA resulted in an increase in intracellular GSH levels. MDA is a marker of lipid oxidation. The increase of MDA level observed in the diabetic control group in this study might be due to the poor antioxidant capacity of mesangial cells as a consequence of low GSH levels [11,13]. Vitamin C and Vitamin E are natural antioxidants *in vivo*. The decreased levels of these antioxidants in the diabetic control group was also an indication of increased oxidative stress in these kidneys. ALA improved the anti-oxidation ability in rats by regenerating vitamin C, vitamin E and SOD through an O-GlcNAc-dependent mechanism [12,14]. In this study, we found a decreased level of SOD in the diabetes control group which was possibly as a result of decreased anti-oxidation capabilities. Moreover, correlation analyses demonstrated that the UPE was negatively correlated with SOD, GSH, and vitamin E. SG and SM were significantly negatively associated with SOD. This suggests that the oxidative stress in kidneys of diabetics leads to impaired renal function [11,12].

This study has found that the kidney weight and the kidney/body weight ratio of the diabetic control group increased significantly compared to the normal control group, which indicated that the increase of kidney weight was possibly caused by some associated pathological changes. They were observed by light and electron microscopy in terms of glomerular hypertrophy, expansion of the mesangial region, mesangial cell proliferation, thickening and interruptions in the GBM, deposition of glycogen, and increased collagen fiber, *etc.* These findings indicate the presence of early glomerulosclerosis in the diabetic kidney however treatment with ALA may ameliorate this glomerulosclerosis [17].

p22phox and p47phox, the NADPH oxidase subunits in the cytomembrane and cytoplasm, are primarily expressed in mesangial cells in kidney. Mesangial cells in a hyperglycemia environment express more NADPH oxidase, which leads to increased ROS in the kidney [16,17,19]. Previous studies have demonstrated that one of the mechanisms contributing to increased oxidative stress in the diabetic kidney is increased expression of NADPH oxidase subunits [16,17,19]. The inhibition of NADPH oxidase resulted in the amelioration of renal histomorphologic changes and impaired function [19]. The data in this study also demonstrated that MDA levels were positively correlated with p22phox and p47phox, and ALA administration decreased the diabetes-associated up-regulation of p22phox and p47phox expression. Thus, this study clearly indicates that ALA reduced oxidative stress by regulating the overexpression of NADPH oxidase involved in the formation of ROS in diabetic kidneys.

As a nuclear factor with multidirectional regulation, NF-κB regulates the expression of inflammatory factors. In this study the expression of NF-κB increased significantly in the diabetic control group compared to the normal control group. There were significant positive correlation between p22phox, p47phox and NF-κB. This indicates that the increased ROS in the kidney leads to an increased expression of NF-κB. Increased expression of NF-κB may activate TGF-β1, which results in increased synthesis of matrix proteins such as collagen type IV, fibronectin, and laminin [20,21]. Moreover, TGF-β1 inhibits the degradation of ECM, and accelerates the accumulation of ECM in the glomerular mesangium and expands the mesangial region. In this study, using electron microscopy the most obvious changes were both thickening of the glomerular basement membrane and increase in the ECM in the diabetic control group. The increased ECM is thought to be primarily due to the increased activation of NF-κB [20]. We found that the ALA-treated group expressed a relatively lower level of NF-κB. This may be the reason that the ALA-treated group demonstrated less serious morphologic changes compared to the diabetic control group.

This study also showed that changes in podocyte structure that occur during the development of diabetic nephropathy. The podocyte is an integral part of the filtration barrier, and changes in their structure have been observed in a broad range of proteinuric glomerular diseases such as diabetes. Podocytes are considered to have limited capacity to replicate post-natally [22]. Thus, the loss of podocytes destroys the structure of the glomerular basement membrane. The foot processes of the podocytes may widen which results in a reduction in the ability of the podocytes to remain attached to the glomerular basement membrane. The consequent areas of bare glomerular basement membrane could result in glomerulosclerosis [23]. This study demonstrated that these changes in the foot processes had been ameliorated after 12 weeks treatment with ALA, suggesting that oxidative stress may be one of the causes of the injury to podocytes [23].

There was limitation in this study. We have only measured mRNA expression of p22phox and p47phox, rather other NADPH oxidase subunits such as Nox4 without protein level assessment.

## 4. Methods

Goto-Kakisaki (GK) rats are characterized by spontaneously elevated blood glucose and are widely used as a model of type 2 diabetes. They are homogenetic with Wistar rats. Ten male GK rats aged eight weeks provided by the Animal Institute from the Chinese Academy of Science were randomly divided into two groups: the diabetic control group (*n* = 5) and the ALA treatment diabetic group (*n* = 5). Four healthy male Wistar rats served as a normal control group. All rats were housed in cages (two to three rats per cage) in a room maintained at 21 ± 1 °C, 45%–50% humidity, and 12 h light-dark cycle. All animals were given water and food composed of 80% regular bait vessel, 10% axungia porci, and 10% white sugar ad libitum. All rats were also weighed at 2 week intervals. Rats in the ALA treatment group were administered ALA 35 mg/kg disolved in saline via intraperitoneal injection QOD for 12 weeks (Lot Number X20000466, Stada, Germany). The rats in the other two groups were injected with saline as a placebo. The local ethics committees approved this study.

### 4.1. Blood Glucose Evaluation

Blood samples from tail veins were determined every 2 weeks for 12 weeks. Blood glucose was measured by Glucometer (Roche Pharmaceuticals & Chemicals Ltd, Basel, Switzerland) using the glucose oxidase assay.

### 4.2. Urine Protein Excretion (UPE)

At the end of the 12th week, all rats were transferred into metabolic cages to collect 24-h urine samples for the measurement of urine protein excretion (Automatic biochemical analysis equipment, MODULAR P800, Roche Diagnostics, Switzerland).

### 4.3. Morphology and Realtime Reverse Transcriptase (RT)-PCR

All rats were anesthetized with sodium pentobarbital (40 mg/kg intraperitoneally). After perfusion with ice-cold saline to achieve freedom of blood in situ, the kidneys were removed bilaterally. Every left kidney was weighed. A 1 mm^3^ mass was removed from the cortex of every right kidney and drenched into 2.5% glutaraldehyde for electron microscopy (Phillip TECNAI 12, Amsterdam, Holland) microanalysis to assess ultraminiature changes and to measure the thickness of the glomerular basement membrane (GBM).

The second mass of right kidney cortex was frozen in liquid nitrogen for measurement of mRNA expression of NF-κB and NADPH oxidase subunits p22phox and p47phox via realtime RT-PCR. RNA was extract with Trizol agent from tissue homogenate. Oligo-primed first strand cDNA synthesis was carried out on total RNA (1 μg) with a reverse transcription system using reverse transcriptase (Promega, Madison, WI, USA). The reaction was incubated at 37 °C for 60 min, heated to 95 °C for 5 min and then quick-chilled on ice. A mixture of 2.5 μL PCR buffer, 3 μL 2.5 mM MgCl_2_ solution, 0.5 μL 20 mM PCR primer, 3 μL 2.5 mM dNTP solution (containing 2.5 mM dATP, dGTP, dCTP and dTTP respectively), 3 units Tag pclymerase (the above all purchase from Promega, Madison, WI, USA), 1 μL cDNA and 14.5 μL distilled water was put to PCR reaction on the PCR equipment (Rotor-Gene 3000 Realtime PCR equipment, Corbett Research, Sydney, Australia). The relative amount of each target gene was achieved by calculating the ratio of the RNA expression of target gene and βactin. The primers of each target gene were:

B-actin: 5′CCTGTACGCCAACACAGTGC3′, 5′ATACTCCTGCTTGCTGATCC3′;
p22phox: 5′GGACGCTTCACGCAGTGGTA3′, 5′GGACAGCAGTAAGTGGAGGACA3′;
p47phox: 5′ATGGGACTGCCCGTGAAGAT3′, 5′GGATGATGGGACCCGTGATG3′;
NF-κB: 5′ACTGCCGGGATGGCTTCTAT3′, 5′CTGGATGCGCTGGCTAATGG3′.

The left kidney was drenched in 4% paraformaldehyde, embedded in paraffin, and sectioned at 6 μm for optical microscopy and morphologic analysis. The sections were stained with hematoxylin and eosin (H&E), periodic acid-Schiff (PAS) for demonstration of glycogen content, and Masson’s trichrome stain for demonstration of collagen deposition.

Sections were examined using an Olympus light microscope (Olympus CX41, Tokyo, Japan). Five sections including 10 glomeruli per section were randomly selected in which to measure the area per glomeruli and mesangial region (Motic Image Advanced 3.1 Image Analysis System, Motic, Xiamen, China). The same method was applied to the Masson’s-stained sections to determine the degree of glomerulosclerosis via a semi-quantitative scoring method as previously described. This method involved evaluating the degree of green stain (represents the degree of fibrosis) and assigning a value between 0 and 4. 0 represented normal tissue, 1 represented glomerulosclerosis in 25% of glomeruli, 2 represented 25%–50% glomerulosclerosis, 3 represented 50%–75% glomerulosclerosis, and 4 represented >75% glomerulosclerosis.

### 4.4. Biochemical Analysis

The remaining tissue from the right kidneys of all rats was placed in ice-cold 0.9% NaCl in a proportion of 1:9 and homogenized using a mechanical homogenizer. After centrifuging the homogenate at 3000 rpm for 15 min, the supernatant was removed and used to measure malonaldehyde (MDA), vitamin C, vitamin E, glutathione (GSH) and superoxide dismutase (SOD). These parameters were assayed according to the instructions accompanying the kits. All chemicals and reagents were purchased from JIANCHENG Bioengineering Institute, NanJing, China.

### 4.5. Statistical Analysis

Data were analyzed using the SPSS13.0 statistical package. Results were expressed by the arithmetic mean ± SD. Differences between groups were analyzed using the ANOVA LSD test. Pearson correlation coefficient (*r*) was used to study associations between variables. A two-tailed test was used in all analyses. The level of significance was set at α = 0.05.

## 5. Conclusions

Diabetic nephropathy develops secondary to a combination of increased oxidative stress and a decreased antioxidant ability of the kidney. Treatment with antioxidants such as ALA can reduce oxidative stress and improve the antioxidant ability of the organ. ALA as an antioxidant can prevent diabetic nephropathy.

## Figures and Tables

**Table 1:** Effects of α-lipoic acid on body and kidney weight and blood glucose.

	Normal control	Diabetic control	ALA-treated diabetic
Blood glucose (mmol/L)	4.2 ± 0.4	8.7 ± 4.0 *	9.4 ± 2.1 *
Body weight (g)	436.3 ± 83.8	305.8 ± 8.4 **	327 ± 19.6 **
Kidney weight (g)	0.7 ± 0.0	1.5 ± 0.1 **	1.2 ± 0.1 **Δ
Kidney/body weight (%)	0.16 ± 0.03	0.50 ± 0.03 **	0.41 ± 0.08 *Δ

Data are expressed as mean ± SD;

* *p* < 0.05,

** *p* < 0.01 *vs.* normal control group;

Δ *p* < 0.05 *vs.* diabetic control group.

**Table 2:** Effects of α-lipoic acid on renal function and structure.

	Non control	Diabetic control	ALA-treated diabetic
UPE (mg/24 h)	8.5 ± 6.1	12.0 ± 3.8 *	9.7 ± 3.8
Glomerulosclerotic index	1.25 ± 0.50	2.50 ± 0.58 *	2.0 ± 0.71
S_M_ (μm^2^)	65.6 ± 13.5	209.9 ± 77.0 **	99.5 ± 32.8 ΔΔ
S_G_ (μm^2^)	844.4 ± 190.7	1573.2 ± 208.7 **	1325.2 ± 320.9 *
S_M_/S_G_ (%)	7.9 ± 1.5	13.4 ± 4.4 *	8.0 ± 3.6 Δ
GBM (μm)	0.25 ± 0.06	0.39 ± 0.11 **	0.29 ± 0.06 ΔΔ

Data are expressed as mean ± SD;

* *p* < 0.05,

** *p* < 0.01 *vs.* normal control group;

Δ *p* < 0.05,

ΔΔ *p* < 0.01 *vs.* diabetic control group.

**Table 3:** The mRNA expression of NF-κB and NADPH oxidase subunit.

	Normal control	Diabetic control	ALA-treated diabetic
NF-κB (×10^−3^)	13.8 ± 5.6	46.9 ± 37.4 *	29.7 ± 6.5
p22phox (×10^−5^)	10.0 ± 1.8	32.9 ± 37.7 *	2.7 ± 1.9
p47phox (×10^−4^)	6.6 ± 4.3	19.5 ± 17.4 *	8.6 ± 2.4

Data are expressed as mean ± SD;

* *p* < 0.05 *vs.* normal control group.

**Table 4:** Quantitation of glutathione (GSH), superoxide dismutase (SOD), Vitamin E, Vitamin C and malonaldehyde (MDA) levels in kidneys.

	Normal control	Diabetic control	ALA-treated diabetic
GSH (mg/mgprot)	74.7 ± 2.0	63.6 ± 4.4 **	69.4 ± 4.8 Δ
SOD (U/mgprot)	9.3 ± 5.5	0.5 ± 0.2 **	4.8 ± 1.7 Δ
VE (μg/mgprot)	0.09 ± 0.02	0.04 ± 0.01 **	0.06 ± 0.01 Δ
VC (μg/mgprot)	1.8 ± 0.2	1.4 ± 0.1 **	1.7 ± 1.2 Δ
MDA (nmol/mgprot)	1.3 ± 0.2	1.7 ± 0.4	1.1 ± 0.3 Δ

Data are expressed as mean ± SD;

** *p* < 0.01 *vs.* normal control group;

Δ *p* < 0.05 *vs.* diabetic control group.
